# Supplementary material for: SnS2 Nanosheets with RGO Modification as High-Performance Anode Materials for Na-Ion and K-Ion Batteries
Source: Nanomaterials (Basel). 2021 Jul 27;11(8):1932. doi: 10.3390/nano11081932 (PMC8401318; doi:10.3390/nano11081932)
Supplement: Supplementary file 1 [file nanomaterials-11-01932-s001.zip › nanomaterials-1322414-supplementary.pdf]

nanomaterials
MDPI

Supplementary Materials

SnS_2 Nanosheets with RGO Modification as High-Performance Anode Materials for Na-ion and K-ion Batteries

Leqiang Wu, Hengjia Shao, Chen Yang, Xiangmin Feng, Linxuan Han, Yanli Zhou, Wei Du, Xueqin Sun, Zhijun Xu, Xiaoyu Zhang, Fuyi Jiang * and Caifu Dong *

School of Environmental and Material Engineering, Yantai University, Yantai 264005, China; wlq_246@163.com (L.W.); shao0331@163.com (H.S.); yc083625@163.com (C.Y.); fxm18763285297@163.com (X.F.); a13156280283@163.com (L.H.); zhouyanli@ytu.edu.cn (Y.Z.); duwei@ytu.edu.cn (W.D.); sxq@ytu.edu.cn (X.S.); xuzhijun@ytu.edu.cn (Z.X.); zhangxiaoyu@ytu.edu.cn (X.Z.)
* Correspondence: fyjiang@ytu.edu.cn (F.J.); dongcf@ytu.edu.cn (C.D.)

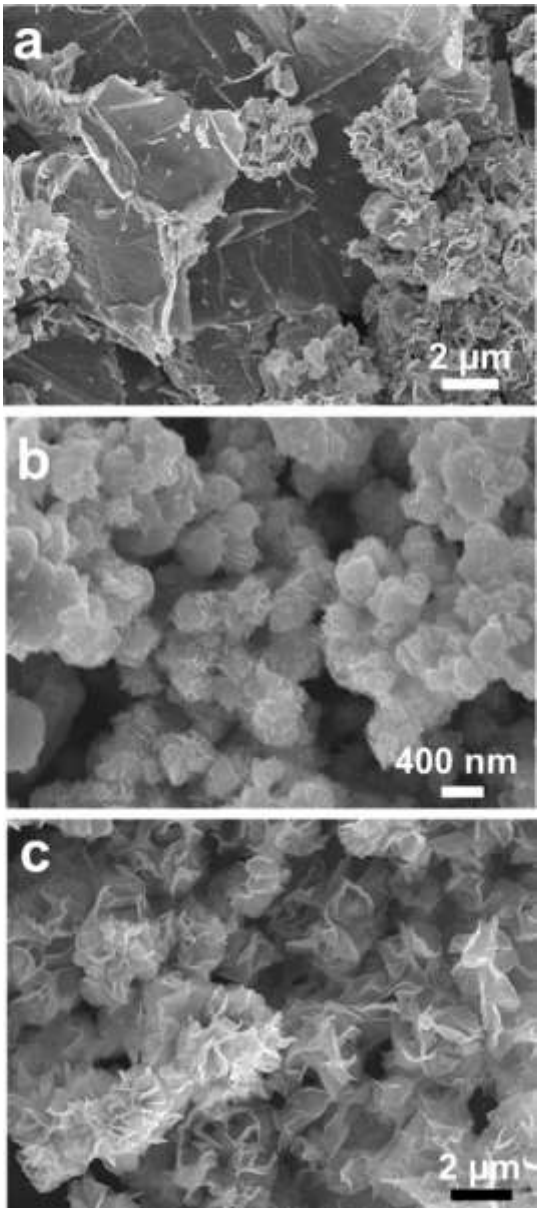

Figure S1. FESEM images of the precursors obtained under different reaction conditions. (**a**) In the presence of GO but without 2-MIN. (**b**) In the presence of 2-MIN but without GO. (**c**) In the absence of 2-MIN and GO.

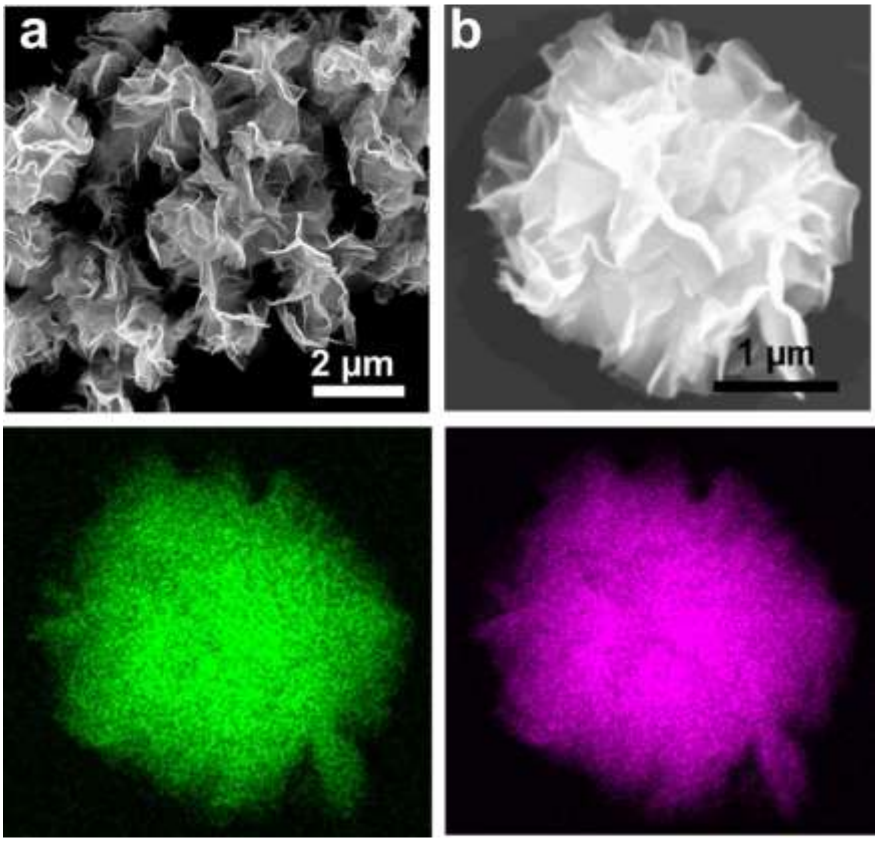

Figure S2. (**a**) FESEM image of SnS_2. (**b**) FESEM image of SnS_2 and the corresponding elemental mappings of Sn and S elements (as labeled).

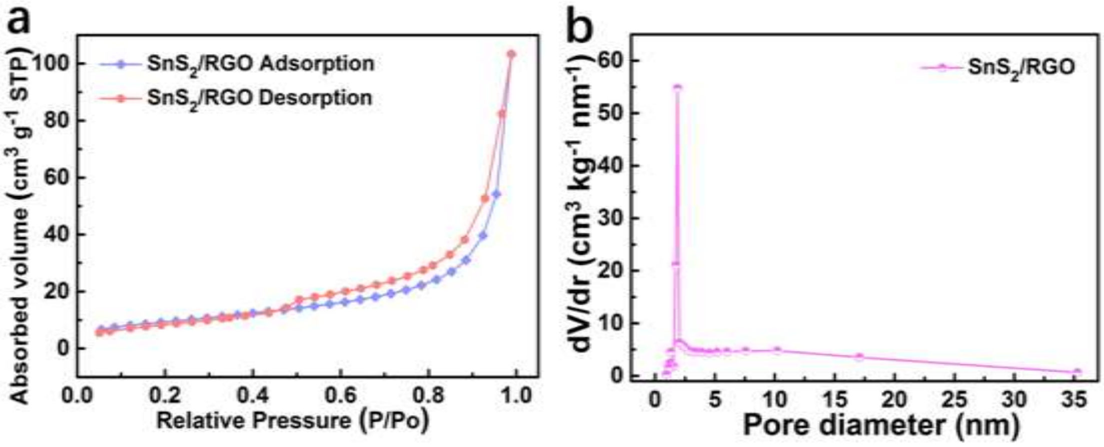

Figure S3. (**a**) Nitrogen adsorption-desorption curve of SnS_2/RGO, (**b**) the related pore size distribution curve.

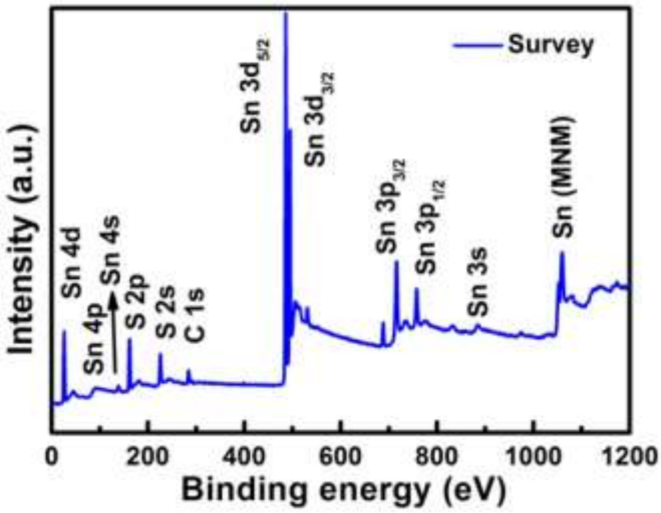

Figure S4. High resolution XPS spectrum of SnS_2.

Table S1. Electrochemical performance comparisons of SnS_2/RGO electrode with those of the previously reported transition metal dichalcogenides anodes for SIBs.

Electrode	Battery	Current Density (mA g^{-1})	Cycle Number	Capacity (mAh g^{-1})	Ref/year
SnS2 Microspheres	SIBs	0.5	100	548	1/2020
GNS/SnS2	SIBs	100	200	462	2/2020
SnS2/3DG	SIBs	100	50	430	3/2020
SnS2@GNS	SIBs	500	100	374.8	4/2020
SnS/RGO	SIBs	100	200	443.4	5/2021
SnS-SnS_2	SIBs	100	200	290.1	6/2019
SnS2-CM	SIBs	200	200	322.4	7/2021
SnS2 NSA	SIBs	500	100	420	8/2017
2D MXene/SnS 2	SIBs	100	200	332	9/2018
SSC@SnS 2	SIBs	100	100	564	10/2018
SnS 2 /S-rGO	SIBs	100	83	530	11/2017
SnS 2 @N,S-GA	SIBs	50	100	360	12/2017
SnS-ZnS@C	SIBs	100	100	585	13/2018
**SnS_2/RGO**	SIBs	500	80	581	**Our work**

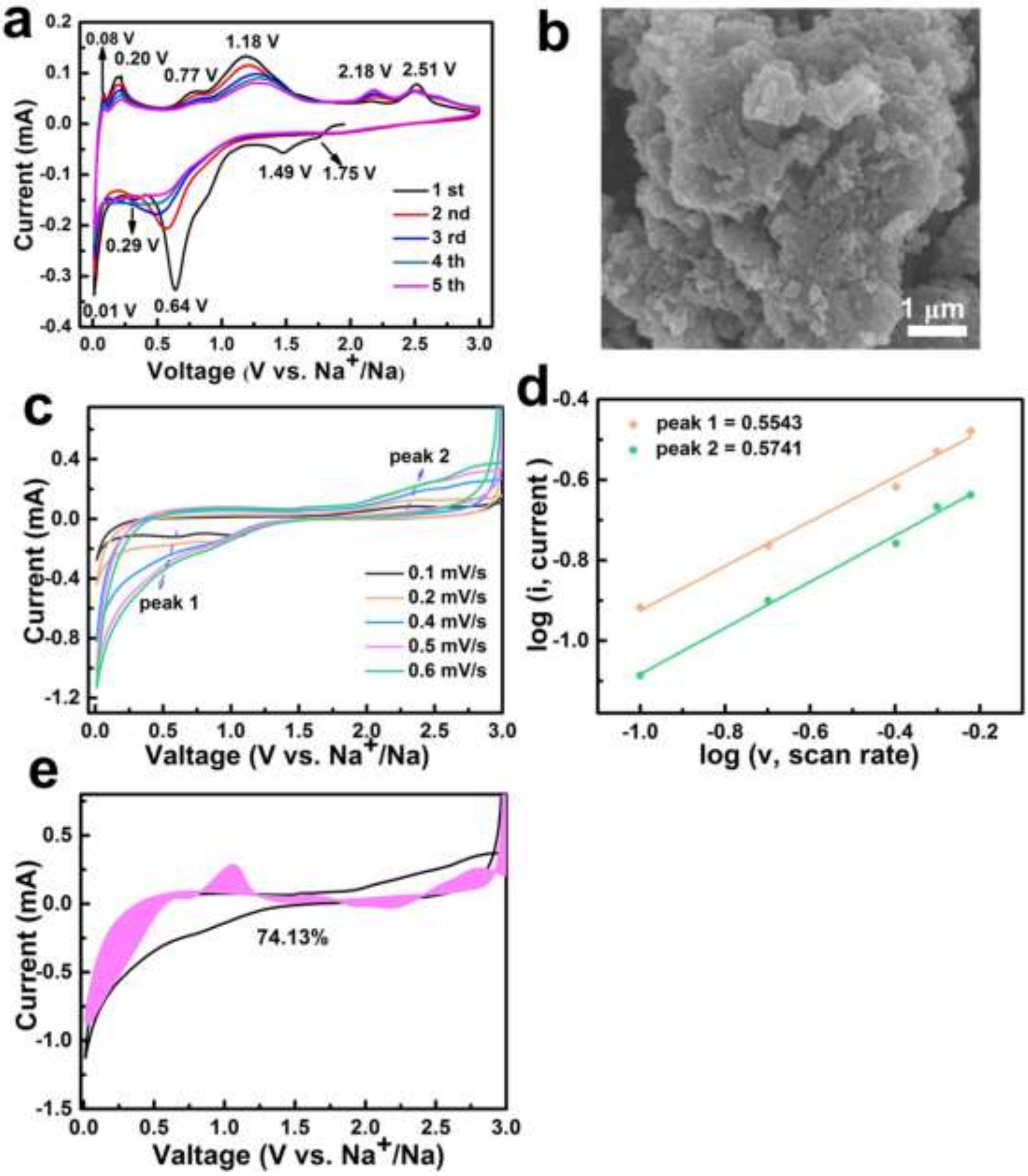

Figure S5. (**a**) Cyclic voltammetry (CV) curves of SnS_2/RGO. (**b**) FEEM image of SnS_2/RGO electrode after rate performance. (**c**) CV profiles of SnS_2 at different sweep rates. (**d**) Linear relationship of log (i) vs. log (v) plots at each redox peak. (**e**) CV curve with capacitive and diffusion-controlled contributions at 0.6 mV s^{-1}, in which the pseudocapacitive fraction is shown in the pink region.

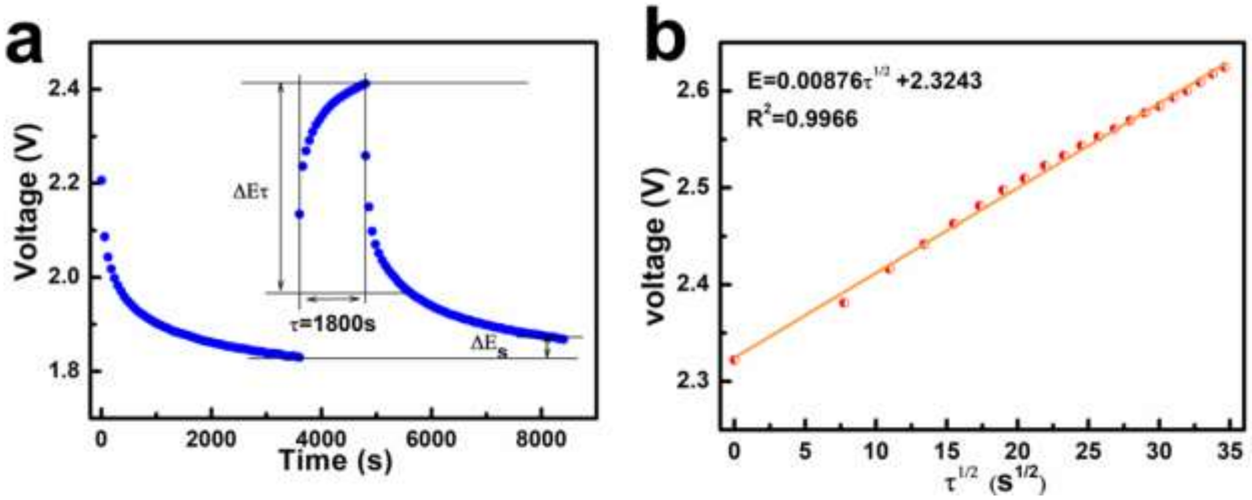

Figure S6. (**a**) Voltage versus time curve for one single GITT test. (**b**) The plots of voltage *vs.* root of pulse time ($\tau_{1/2}$).

During the GITT test in the first cycle, the cell was charged or discharged at 0.1 A g^{-1} for 30 min, then followed by a 90 min open circuit step to allow relaxation back to equilibrium. The procedure was repeated until the discharge (or charge) voltage reached 0.01 V (3 V). Figure S5a shows a single GITT titration curve during the charge process. Where, τ is the relaxation time (s); ΔE_s stands for the voltage change in two consecutive relaxation period; ΔE_τ represents the voltage change during the constant current pulse, regardless of the IR-drop. Figure S5b presents the plot of voltage vs $\tau^{1/2}$ is roughly linear, so the Na^+ diffusion coefficients can be calculated according to the following Equation:

$$D_{\mathrm{Na}} = \frac{4}{\pi\tau}\left(\frac{m_B V_m}{M_B S}\right)^2\left(\frac{\Delta E_s}{\Delta E_\tau}\right)^2 \quad (1)$$

D_{Na} (cm^2 s^{-1}) is the chemical diffusion coefficient of Na^+; m_B (g) is the quality of the active material in the electrode; M_B (g mol^{-1}) is the molecular weight of active material; V_m (cm^3 mol^{-1}) is the molar volume of the active material; S (cm^2) is the contacting area of eletrode with electrolyte (taken as the geometric area of electrode for better comparison with literatures); It shows that the D_{Na} values during the insertion and extraction processes are close and on the order of $10^{-10.5}$ to $10^{-11.5}$ cm^2 s^{-1} (Figure 5h, 5i), indicating that Na^+ has a high kinetic behavior in SnS_2/RGO electrode.[14,15] The high Na^+ diffusion coefficient may be due to the unique micro- and nanostructure, which can be responsible for high rate performance.

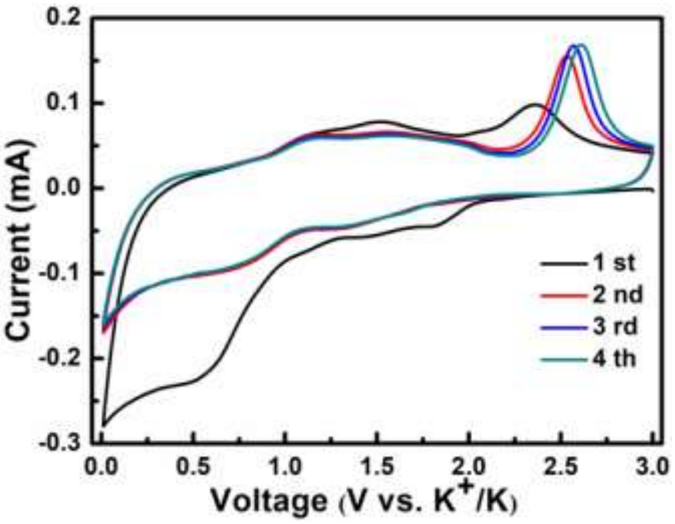

Figure S7. CV curves of the SnS_2/RGO electrode at a scan rate of 0.1 mV s^{-1} for the first four cycles for PIBs.

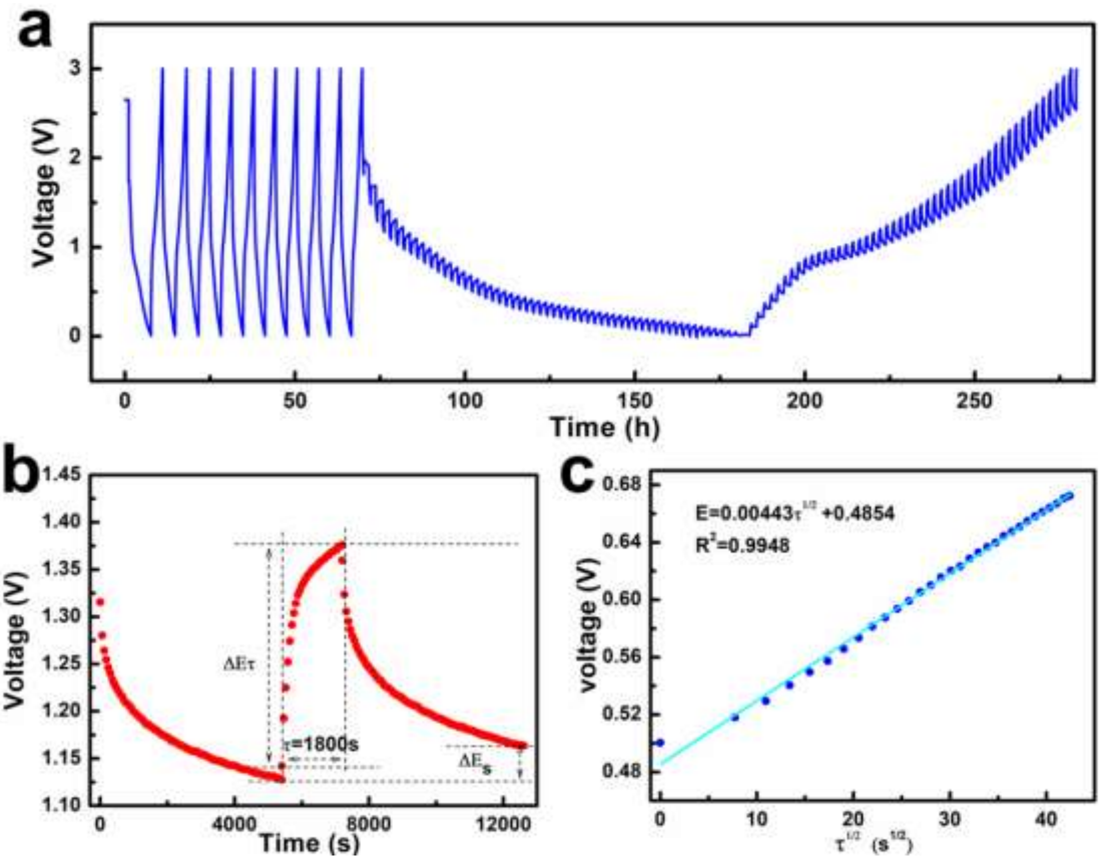

Figure S8. (**a**) The charge/discharge curves of SnS_2/RGO electrode obtained during GITT measurement, (**b**) a single GITT titration curve during the charge process, and (**c**) the plots of voltage vs. root of pulse time ($\tau^{1/2}$).

The GITT test was performed in a voltage range of 0.01–3 V (Figure S7a). Prior to GITT measurement, the assembled cells were charged/discharged at 100 mA g^{-1} for 10 cycles to activate the battery. Figure S7b shows a single GITT titration curve during the charge process. Assuming that K^+ transport in the electrode obeys Fick's second law, the chemical diffusion coefficients can be calculated by the following Equation (2):

$$D_K = \frac{4}{\pi}\left(\frac{m_B v_m}{M_B S}\right)^2\left(\frac{\Delta E_s}{dE_\tau / d\sqrt{\tau}}\right)^2 \qquad \left(\tau \ll \frac{L^2}{D_K}\right) \tag{2}$$

When $dE_\tau / d\sqrt{\tau}$ shows a linear relationship, the equation can be simplified into: $D_K = \frac{4}{\pi\tau}\left(\frac{m_B v_m}{M_B S}\right)^2\left(\frac{\Delta E_s}{\Delta E_\tau}\right)^2$ Where, D_K (cm^2 s^{-1}) is the chemical diffusion coefficient of K^+; m_B (g) is the quality of the active material in the electrode; M_B (g mol^{-1}) is the molecular weight of active material; v_m (cm^3 mol^{-1}) is the molar volume of the active material; S (cm^2) is the contacting area of eletrode with electrolyte (taken as the geometric area of electrode for better comparison with literatures); ΔE_s is the voltage change in two consecutive relaxation period; ΔE_τ is the voltage change during the constant current pulse. It can be seen that the plots of voltage vs $\tau^{1/2}$ are roughly linear (Figure S7c), so the K^+ diffusion coefficients at different discharge and charge states can be calculated according to the simplified Eq. 2.

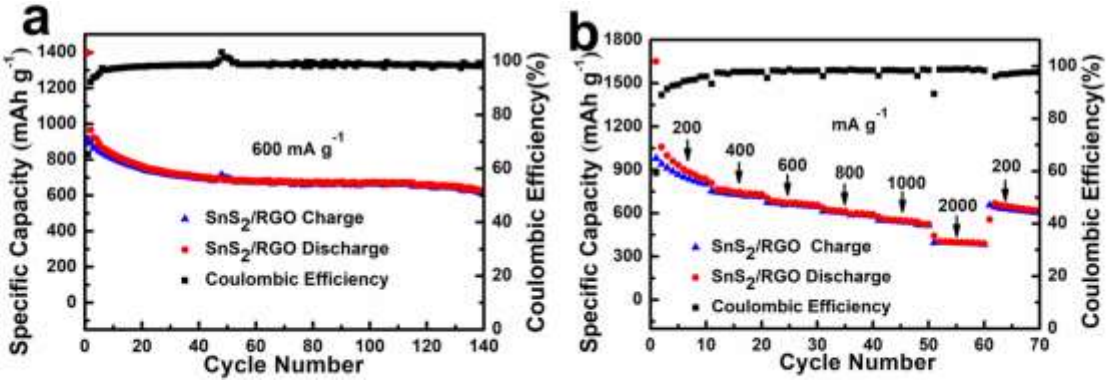

Figure S9. (**a**) The cycling performance of SnS_2/RGO at 0.6 A g^{-1}. (**b**) Rate performance of SnS_2/RGO electrode at various densities of 0.2–2.0 A g^{-1}.

References

1. Wang, J.; Huang, J.; Huang, S.; Notohara, H.; Urita, K.; Moriguchi, I.; Wei, M. Rational Design of Hierarchical SnS_2 Microspheres with S Vacancy for Enhanced Sodium Storage Performance. *ACS Sustainable Chem. Eng.* **2020**, *8*, 9519–9525.
2. Sang, Z.; Yan, X.; Su, D.; Ji, H.; Wang, S.; Dou, S. X.; Liang, J. A Flexible Film with SnS_2 Nanoparticles Chemically Anchored on 3D-Graphene Framework for High Areal Density and High Rate Sodium Storage. *Small* **2020**, *16*, 2001265.
3. Zeng, L.; Zhang, L.; Liu, X.; Zhang, C. SnS_2 Nanocrystalline Anchored Three Dimensional Graphene for Sodium Batteries with Improved Rate Performance. *Nanomaterials* **2020**, *10*, 2336.
4. Wu, Y. Q.; Yang, Y.; Pu, H.; Gao, R. Z.; Meng, W. J.; Yang, H. X.; Zhao, D. L. SnS_2 Nanoparticle Integrated Graphene Nanosheets as High Performance and Cyclestable Anodes for Lithium and Sodium Storage. *J. Alloy. Compd*. **2020**, 822, 153686.
5. Mao, W.; Ding, Y.; Li, M.; Ma, C.; Gong, H.; Pan, J.; Zhang, S.; Qian, Y.; Bao, K. Synthesis and Electrochemical Characterization of 2D SnS_2/RGO as Anode Material in Sodiumion Batteries. *J. Alloy. Compd*. **2021**, 855, 157209.
6. Zhang, S. W.; Lv, W.; Qiu, D.; Cao, T.; Zhang, J.; Lin, Q.; Chen, X.; He, Y. B.; Kang, F.; Yang, Q. H. An ion-conducting SnS-SnS_2 Hybrid Coating for Commercial Activated Carbons Enabling Their Use as High Performance Anodes for Sodium Ion Batteries. *J. Mater Chem A*. **2019**, *7*, 10761–10768.
7. Liu, H.; Wei, C.; Ai, Z.; Li, M.; Xu, M.; Ma, C.; Shi, J. The Positive Effect of 3D Interpenetrating Network Porous Structure by Carbon Membranes on Alleviating the Volume Expansion of SnS_2 Nanosheets for Enhancing Lithium and Sodium Storage. *Colloids and Surfaces A: Physicochemical and Engineering Aspects* **2021**, *610*, 125937.
8. Wang, Y.; Zhou, J.; Wu, J.; Chen, F.; Li, P.; Han, N.; Huang, W.; Liu, Y.; Ye, H.; Zhao, F.; Li, Y. Engineering SnS_2 Nanosheet Assemblies for Enhanced Electrochemical Lithium and Sodium Ion Storage. *J. Mater Chem A*. **2017**, *5*, 25618–25624.
9. Wu, Y.; Nie, P.; Wu, L.; Dou, H.; Zhang, X. 2D MXene/SnS_2 Composites as High Performance Anodes for Sodium Ion Batteries. *Chem. Eng. J*. **2018**, *334*, 932938.
10. Yang, Z.; Zhang, P.; Wang, J.; Yan, Y.; Yu, Y.; Wang, Q.; Liu, M. Hierarchical Carbon@SnS_2 Aerogel with "Skeleton/Skin" Architectures as a High Capacity, High Rate Capability and Long Cycle Life Anode for Sodium Ion Storage. *ACS Appl Mater Interfaces* **2018**, *10*, 37434–37444.
11. Zheng, P.; Dai, Z.; Zhang, Y.; Dinh, K. N.; Zheng, Y.; Fan, H.; Yang, J.; Dangol, R.; Li, B.; Zong, Y.; Yan, Q.; Liu, X. Scalable Synthesis of SnS_2/S-doped Graphene Composites for Superior Li/Na Ion Batteries. *Nanoscale* **2017**, *9*, 14820–14825.
12. Fan, L.; Li, X.; Song, X.; Hu, N.; Xiong, D.; Koo, A.; Sun, X. Promising Dual Doped Graphene Aerogel/SnS_2 Nanocrystal Building High Performance Sodium Ion Batteries. *ACS Appl. Mater. Interfaces* **2018**, *10*, 2637–2648.
13. Zhang, Y.; Wang, P.; Yin, Y.; Zhang, X.; Fan, L.; Zhang, N.; Sun, K. Heterostructured SnS-ZnS@C Hollow Nanoboxes Embedded in Graphene for High Performance Lithium and Sodium Ion Batteries. *Chem. Eng. J.* **2019**, *356*, 1042–1051.
14. Xiao,Y. H.; Zhao, X. B.; Wang, X. Z.; Su, D. C.; Bai, S.; Chen, W.; Fang, S. M.; Zhou, L. M.; Cheng, H. M.; Li, F. A Nanosheet Array of Cu_2Se Intercalation Compound with Expanded Interlayer Space for Sodium Ion Storage. *Adv. Energy Mater*., 2020, 2000666.
15. Sun, Z. H.; Wu, X. L.; Xu, J. N.; Qu, D. Y.; Zhao, B. L.; Gu, Z. Y.; Li, W. H.; Liang, H. J.; Gao, L. F.; Fan, Y. Y.; Zhou, K.; Han, D. X.; Gan, S. Y. Y.; Zhang W.; Niu, L. Construction of Bimetallic Selenides Encapsulated in Nitrogen/Sulfur Co-Doped Hollow Carbon Nanospheres for High-Performance Sodium/Potassium-Ion Half/Full Batteries. *Small* 2020, 1907670.
